# Apoptosis Evaluation in Circulating CD34+-Enriched Hematopoietic Stem and Progenitor Cells in Patients with Abnormally Increased Production of Endogenous Glucocorticoids in Course of Cushing’s Syndrome

**DOI:** 10.3390/ijms232415794

**Published:** 2022-12-13

**Authors:** Miłosz P. Kawa, Anna Sobuś, Ewa Pius-Sadowska, Karolina Łuczkowska, Dorota Rogińska, Szymon Wnęk, Edyta Paczkowska, Mieczysław Walczak, Anhelli Syrenicz, Bogusław Machaliński

**Affiliations:** 1 Department of General Pathology, Pomeranian Medical University in Szczecin, 72 Powstancow Wlkp. Street, 70-111 Szczecin, Poland; 2 Department of Pediatrics, Endocrinology, Diabetology, Metabolic Diseases and Cardiology of the Developmental Age, Pomeranian Medical University in Szczecin, 1 Unii Lubelskiej Street, 71-252 Szczecin, Poland; 3 Department of Endocrinology, Metabolic Diseases and Internal Diseases, Pomeranian Medical University in Szczecin, 1 Unii Lubelskiej Street, 71-252 Szczecin, Poland

**Keywords:** glucocorticoids, apoptosis, Cushing’s Syndrome, hematopoietic stem and progenitor cells, microarrays, microRNA, RNA, BCL-2

## Abstract

Abnormalities in hematological parameters of peripheral blood have been noted in patients with endogenous Cushing’s Syndrome (CS) in the corticotropin (ACTH)-dependent and ACTH-independent forms. Nevertheless, the exact mechanism of glucocorticoids (GCs) action on human hematopoiesis is still not entirely clear. The aim of the study was to determine whether endogenous excessive production of GCs could affect apoptosis of CD34+ cells enriched in hematopoietic stem and progenitor cells (HSPCs) collected from the peripheral blood of newly diagnosed CS patients. Flow cytometry, Annexin-V enzyme-linked immunosorbent assay, TUNEL assay, real-time quantitative PCR, and microarray RNA/miRNA techniques were used to characterize CS patients’ HSPCs. We found that the glucocorticoid receptor (GR) protein expression levels in CS were higher than in healthy controls. A complex analysis of apoptotic status of CS patients’ HSPC cells showed that GCs significantly augmented apoptosis in peripheral blood-derived CD34+ cells and results obtained using different methods to detect early and late apoptosis in analyzed cell population were consistent. CS was also associated with significant upregulation in several members of the BCL-2 superfamily and other genes associated with apoptosis control. Furthermore, global gene expression analysis revealed significantly higher expression of genes associated with programmed cell death control in HSPCs from CS patients. These findings suggest that human endogenous GCs have a direct pro-apoptotic activity in hematopoietic CD34+ cells derived from CS subjects before treatment.

## 1. Introduction

Glucocorticoids (GCs) are a class of steroid hormones with multiple functions. GCs regulate function of different human organs, including the brain, liver, muscle, and bones. GCs also exert immunoregulatory effects. Indeed, sustained hypercortisolism leads to specific changes in the number of white blood cells (WBC) circulating in peripheral blood, a general increase in the total WBC count, and elevated neutrophil and monocyte numbers. The relation of an elevated WBC count with long-term excessive GC exposure was later shown to be reversible following successful treatment in patients with endogenous Cushing’s Syndrome (CS) [1]. On one hand, it was observed that glucocorticoid-induced granulocytosis could be the effect of demargination of intravascular granulocytes [2]. On the other hand, it was proposed that GCs may inhibit apoptosis of human mature neutrophils, thus increasing neutrophil survival in human body [3]. Furthermore, the robust anti-inflammatory and immunosuppressive properties of long-term GC exposure remain due to potential effects on immune cell functions, including decreasing several proinflammatory cytokines expression, increasing production of immunosuppressive proteins, together with inducing the pro-inflammatory lymphocyte apoptosis in peripheral blood [4]. An additional mechanism for GC anti-inflammatory and immunosuppressive effects was the observed potential to inhibit intercellular adhesion of lymphocytes to endothelium binding sites through the down-modulation of lymphocyte adhesion molecules [5]. The above observations confirmed the association between glucocorticoids and leucopoiesis and suggest an important role of direct GC control in the expression of the corresponding genes in different mature white blood cells circulating in peripheral blood. However, some other studies, and our previous observations [6,7,8], have demonstrated a direct role of different hormones in non-physiological concentrations on human hematopoietic stem cells, which may also partly explain the mechanisms of quantitative abnormalities in the numbers of lineage-committed mature blood cells observed in the peripheral blood of patients with different endocrine diseases.

Cushing’s disease, known as “central Cushing’s Syndrome”, is a life-threatening neuroendocrine disorder caused by a pituitary adenoma, which leads to excess ACTH secretion and adrenal-derived cortisol [9]. In addition, there are patients with “adrenal Cushing’s Syndrome”, caused by unilateral adrenal tumors, including adenomas and carcinomas [10]. Rarely, Cushing’s Syndrome is caused by primary bilateral macronodular adrenal hyperplasia or primary pigmented nodular adrenocortical disease and its non-pigmented variant: isolated micronodular adrenocortical disease [11]. Both types of Cushing’s Syndrome (CS) result in excessive adrenal cortisol secretion which binds to the glucocorticoid receptors (GRs), leading to increased morbidity and mortality [12]. Importantly, modern data suggest that the clinical effects of GC exposure on peripheral tissues are determined not only by the amount of the adrenal GC production but also by the peripheral GC metabolism [13,14] and by the GC sensitivity [15]. Therefore, patients with Cushing’s Syndrome need complex and adequate investigations that determine multi-organ effects and consequences of exposure to excessive glucocorticoids.

Regulation of important post-transcriptional regulators such as microRNA (miRNAs) has an essential impact on modulating fundamental cellular processes, including gene expression, cell cycling, cell proliferation, and apoptosis [16,17]. Steroid and peptide hormones appear to act partially through regulating miRNA networks, with important implications in metabolic homeostasis and human metabolic diseases at the molecular level [18,19,20]. Indeed, GC signaling is related to the function of miRNAs, which has been extensively studied for their post-transcriptional involvement in the regulation of gene expression [21]. It was observed that miRNAs can function as direct regulators of GRs via the hybridization of various miRNAs with the 3′untranslated region (3′UTR) of the GR transcript [22]. Moreover, microRNAs are directly or indirectly impacted by GCs, as it has been reported that the expression level of miRNAs is strongly dependent on endogenous GC levels [23]. Furthermore, microRNAs may regulate the expression of GRs and diverse signal transduction-related molecules, interfering with the GC signaling pathways in different types of cells [24].

However, little information is available about the impact of GCs on survival and programmed death of hematopoietic stem and progenitor cells (HSPCs) circulating in peripheral blood of patients with Cushing’s Syndrome, where these cells have been exposed in vivo to GCs in a pathological context of chronic hypercortisolism rather than by artificial treatment with exogenous GCs. In the present study, we investigated the in vivo effects of GCs on apoptosis in CD34+ cells enriched in HSPCs collected from patients with newly diagnosed Cushing’s Syndrome, either central CS or adrenal CS. Using this approach, we will be able to demonstrate whether hematopoietic stem and progenitor cells are prone to GC-induced apoptosis in vivo as well as whether this biological effect is relevant in the context of chronic abnormal concentration of GCs in course of CS in humans. Based on these data our studies will provide deeper insights into the in vivo regulation of human haematopoiesis by GCs. Therefore, the CD34+ cells were examined by different methods for apoptosis activity, including Annexin-V labeling, terminal deoxynucleotidyl transferase-mediated dUTP labeling (TUNEL), and analysis of the expression of apoptosis-related molecules, i.e., BAX, BCL-2, and BCL-xL. Finally, the global gene expression profiles in collected CD34+ cells were analyzed using RNA/miRNA microarrays.

## 2. Results

### 2.1. Characteristics of the Human Subjects

In total, 68 subjects were evaluated. The Cushing’s Syndrome (CS) and healthy control groups were matched for age and gender as well as well-known cardiovascular risk factors, including hypertension, history of ischaemic heart disease, myocardial infarct, cerebral stroke, peripheral artery disease, and aortic aneurysm. There were no significant differences in clinical characteristics between the study groups except for the diagnosed hypercortisolism in the CS group.

### 2.2. GRα Is Expressed at the Protein Level in CD34+ Hematopoietic Cells from CS Patients

**Figure 1:**
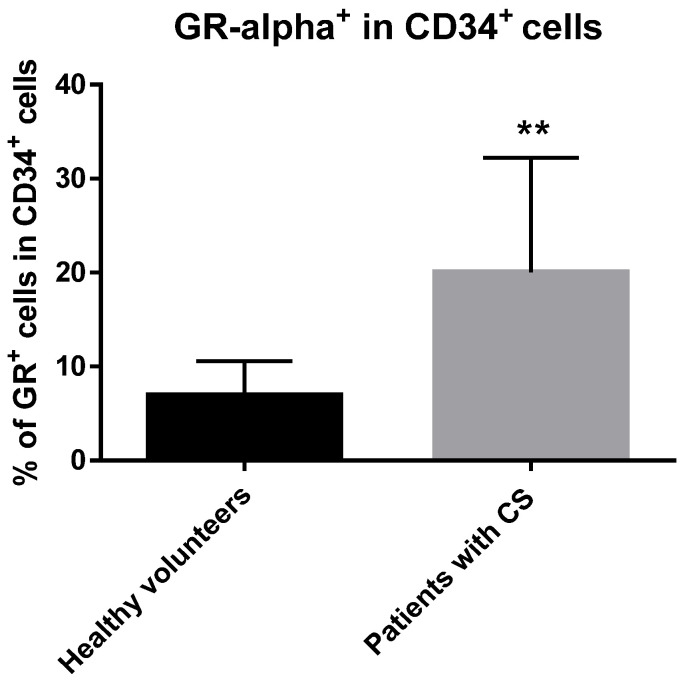
GRα expression in CD34+ cells from PB of patients with CS. ** *p* < 0.01 vs. control group.

To detect GR alpha intracellular protein expression in CD34+ cells, flow cytometry (FC) analysis was performed. CD34+ cells from CS patients and healthy subjects expressed GRα protein as shown in Figure 1. Interestingly, we observed that GR alpha positive cells were significantly increased in CS patients compared to their healthy controls. The hematopoietic origin of isolated CD34+ cells was confirmed by detection of the surface expression of a particular hematopoiesis-related antigen, CD45. These results demonstrate that GRα is expressed in human CD34+ cells from peripheral blood and they are potentially active; thus, the intracellular signal transduction pathways can be induced through binding of the endogenous glucocorticoids in the course of CS.

### 2.3. Glucocorticoids Induce Apoptosis in CD34+ Hematopoietic Cells from CS Patients

**Figure 2:**
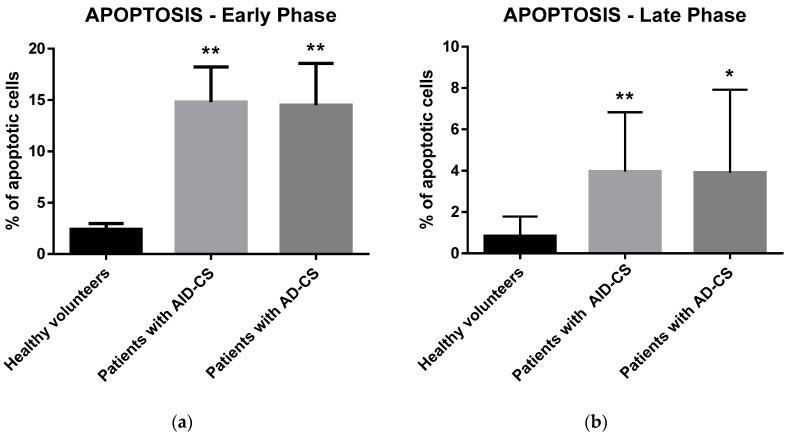
Detection of early and late phase of apoptosis in CD34+ cells from CS patients. The percentage of CD34+ cells in the early (**a**) and late (**b**) phase of apoptosis was evaluated in control subjects and CS patients separated due to the cause of the endogenous glucocorticoid overproduction (AID-CS vs. AD-CS). The results are expressed as the mean value ± S.D. * *p* < 0.05; ** *p* < 0.01 vs. control subjects.

To assess the potential in vivo influence of GCs on apoptosis in CD34+ cells, two different methods were employed: (i) analysis of the early apoptotic stage characterized by Annexin V, which binds to phosphatidylserine on the cell surface; and (ii) the TUNEL assay, which specifically detects the fragments of damaged DNA in cell nuclei as a result of the late apoptotic stage. Annexin V staining revealed a significant increase in the percentage of cells undergoing the early apoptosis phase in CS patients (Figure 2a). We confirmed these data by analyzing the DNA fragmentation intensity, which demonstrated a significant decrease in the percentage of cells undergoing the late apoptosis phase in CS patients (Figure 2b). Interestingly, there were no significant differences between CS patients suffering from ACTH-independent (AID-CS) and ACTH-dependent (AD-CS) causes of endogenous glucocorticoid overproduction (Figure 2a,b).

### 2.4. Glucocorticoids Modulate the Gene Expression of Selected Apoptosis-Regulating Proteins in CD34+ Hematopoietic Cells from CS Patients

**Figure 3:**
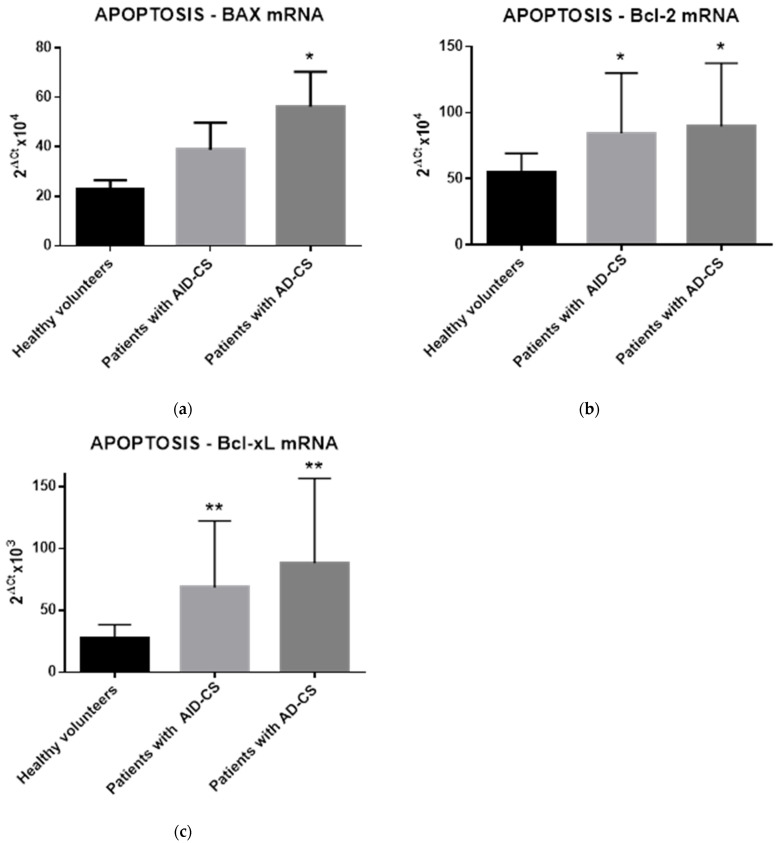
The expression profile of selected apoptosis-related molecules *BAX* (**a**), *BCL-2* (**b**), and *BCL-xL* (**c**) in CD34+ cells from CS patients. The expression of selected apoptosis-related gene transcripts s was evaluated in CD34+ cells of controls and CS patients separated due to the cause of the endogenous glucocorticoid overproduction (AID-CS vs. AD-CS). The results are expressed as the mean value ± S.D. * *p* < 0.05; ** *p* < 0.01 vs. control subjects.

Next, to analyze the expression levels of pro- and anti-apoptotic genes in CD34+ cells collected from all subjects enrolled in the study, we performed a molecular analysis of mRNA expression for the main pro-apoptotic *BAX* gene and two important anti-apoptotic genes, *BCL-2* and *BCL-xL*. Quantitative analysis revealed significantly increased pro-apoptotic as well as anti-apoptotic gene expression in CS patients compared to their controls, as shown in Figure 3. Moreover, we observed even more pronounced expression of mRNA for pro-apoptotic gene *BAX* in patients suffering from ACTH-dependent (AD-CS) compared to ACTH-independent (AID-CS) causes of endogenous glucocorticoid overproduction (nearly 100% upregulation of *BAX* mRNA; *p* < 0.05; Figure 3a). In conclusion, apoptosis is tightly controlled by the balance between pro- and anti-apoptotic members of the BCL-2 protein family. Changes in the relative expression levels of such molecules will ultimately decide the cell fate. To maintain such balance longitudinally both groups of antagonistically acting molecules could be overexpressed at the same time.

### 2.5. Microarray Analysis of Gene Expression Changes in CD34+ Hematopoietic Progenitor Cells from CS Patients

**Figure 4:**
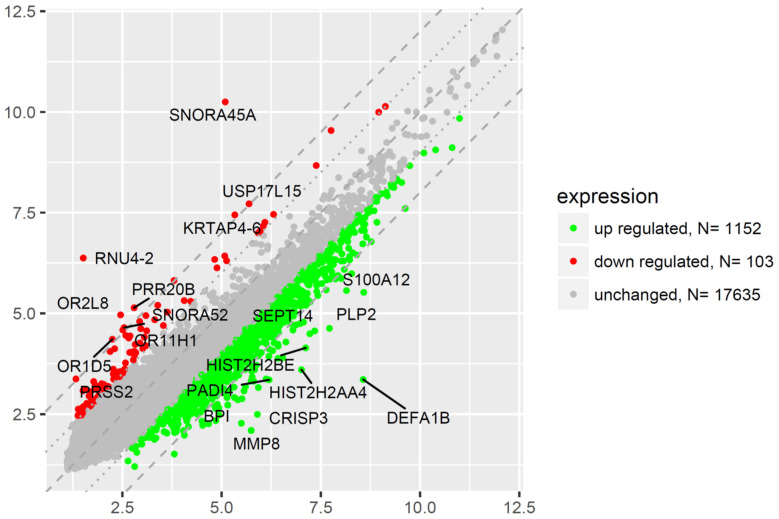
The scatter plot of global gene expression in CD34+ hematopoietic progenitor cells from CS patients when compared to the controls. Red points correspond to downregulated genes (at least two-fold change, *p* < 0.05), green points show upregulated genes (at least two-fold change, *p* < 0.05). The graph also contains names of the genes with the highest change in expression. The graph also contains names of the miRNA with the largest change in expression. On the horizontal axis (*x*-axis) are plotted data from the CS patient group in this study. On the vertical axis (*y*-axis) are plotted data from the healthy subject group, which serves as the control group in this study.

To analyze the mechanism(s) underlying the regulation of the apoptosis process by endogenous GCs, total RNA was isolated from CD34+ hematopoietic progenitor cells collected from the peripheral blood of CS patients and healthy volunteers, and it was subjected to analysis using RNA microarrays containing approximately 18,900 mRNA transcripts. We assumed the following selection criteria for differentially expressed genes (DEG): an expression fold difference > absolute 2 and an adjusted *p*-value ≤ 0.05. Microarray analysis showed that expression of 1152 genes was upregulated at least two-fold and 103 genes were downregulated at least two-fold in CS group compared to the controls (Figure 4). The expression of 17,635 genes was unchanged.

**Figure 5:**
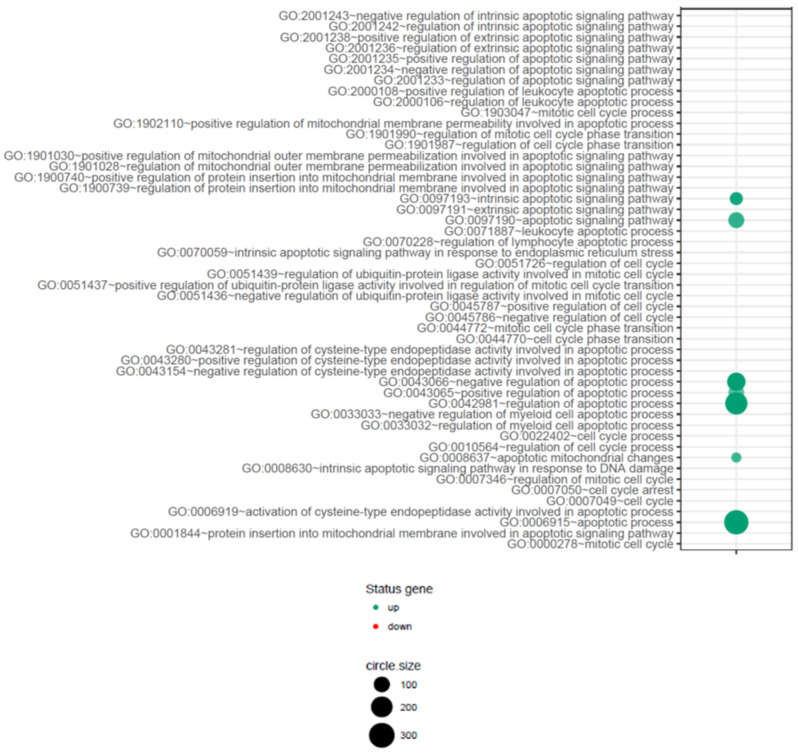
The bubble plot with changed biological processes related to apoptosis, assigned according to Gene Ontology (GO) classifications, in CD34+ hematopoietic progenitor cells from CS patients when compared to their healthy controls. Genes assigned to individual processes fulfilling the criteria of adjusted *p* < 0.05, method = Benjamini, and minimum number of genes per group = 100, are presented. Each bubble size reflects the number of differentially expressed genes represented in the corresponding annotation. The green color represents GO terms where genes are upregulated, while red corresponds to GO terms of downregulated genes. The transparency of the bubbles displays *p*-value (increased transparency is closer to the limit of *p* = 0.05).

Next, we were interested to find out whether differentially expressed genes (DEGs) were involved in control of the apoptosis process; thus, they were classified according to the Gene Ontology (GO) Classification of Biological Process in the relevant ontological groups from GO BP Direct database. We were also interested in which pathway of induction of apoptosis (intrinsic vs. extrinsic) is commonly involved in hematopoietic progenitor cells affected by endogenous GCs. Thus, a whole set of DEGs consisting of 1255 genes was subjected to functional annotation and clusterization using the Database for Annotation, Visualization, and Integrated Discovery (DAVID) bioinformatics tools. Analysis of functional annotations mainly identified upregulated processes (e.g., positive regulation of cellular processes and negative regulation of cellular processes, regulation of cellular metabolic processes, protein metabolic processes and regulation of metabolic processes, cellular response to chemical stimulus, cell death and apoptotic processes). In addition, a detailed analysis of selected pathways related to apoptosis revealed at least seven pathways from GO BP Direct database that are related to apoptosis and its control via positive or negative regulation, including pathways denoted as: intrinsic apoptotic signaling pathway (GO: 0097193); apoptotic signaling pathway (GO: 0097190); negative regulation of apoptotic process (GO: 0043066); positive regulation of apoptotic process (GO: 0046065); regulation of apoptotic process (GO: 0042981); apoptotic mitochondrial changes (GO: 0008637); apoptotic process (GO:0006915) (Figure 5).

**Figure 6:**
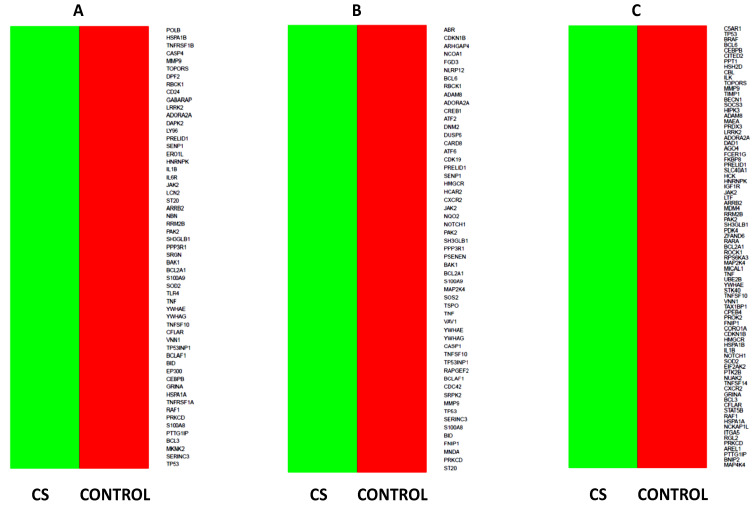

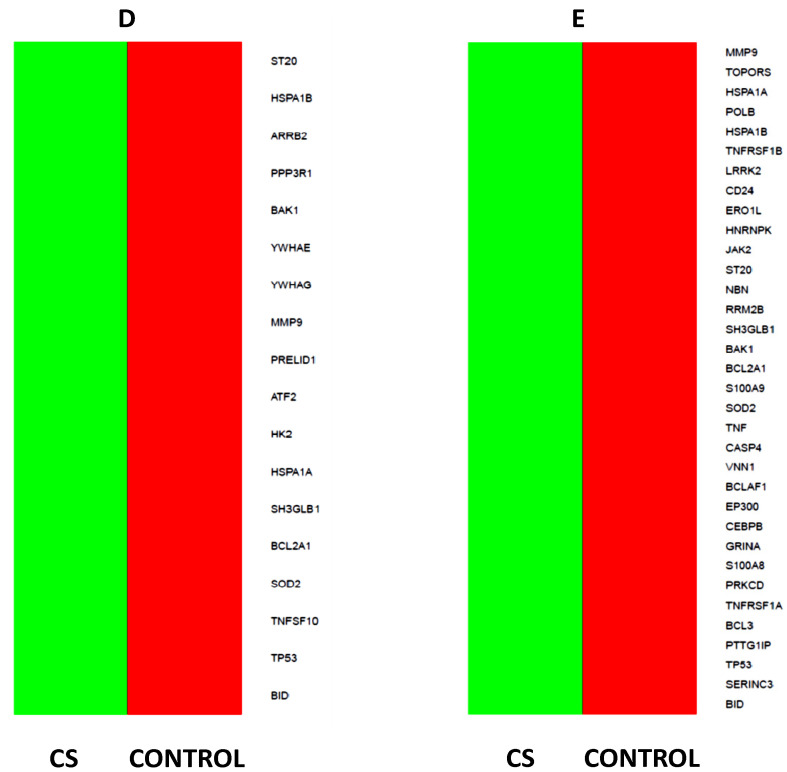
Heatmaps show the dysregulated processes related to apoptosis (Apoptotic signaling pathway (**A**), Positive regulation of apoptotic process (**B**), Negative regulation of apoptotic process (**C**), Apoptotic mitochondrial changes (**D**), and Intrinsic apoptotic signaling pathway (**E**)) and their associated genes in CD34+ hematopoietic progenitor cells from CS patients compared to their healthy controls. Differentially expressed genes are marked by color (upregulated—green, downregulated—red). Colored boxes correspond to genes over the cut-off criteria (|fold| < 1.5, *p* > 0.05).

Differentially expressed genes related to apoptotic process activity and its control are also visualized as heat maps (Figure 6A–E).

**Figure 7:**
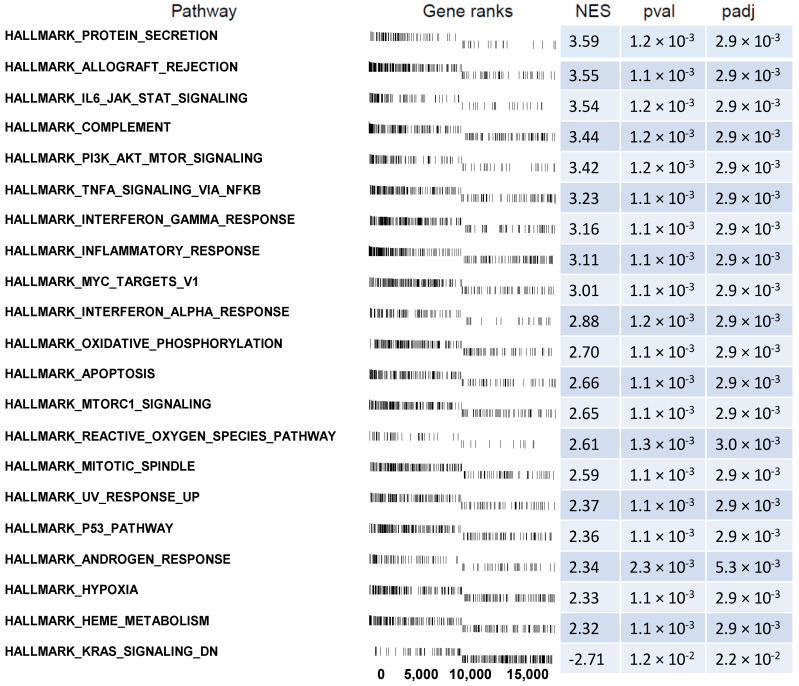
Gene set enrichment analysis in CD34+ hematopoietic progenitor cells from CS patients using hallmark gene sets. A list of significantly enriched gene sets with appropriate gene ranks, normalized enrichment score (NES), *p* values (pval), and *p* values after FDR correction (padj) is displayed.

The above results were confirmed by another powerful bioinformatics tool, Gene Set Enrichment Analysis (GSEA). The GSEA method focuses on gene sets, described as groups of genes that participate in a common biological function, chromosomal location or regulation, etc. Therefore, to more precisely investigate the biological influence of endogenous GCs on CD34+ hematopoietic progenitor cells, we carried out GSEA to identify altered pathways in peripheral blood-derived CD34+ hematopoietic progenitor cells from CS patients vs. control cells. In this analysis, the fold change values of all genes were log2 transformed and ranked according their logFC. Afterwards, these values were used for a 1000 times permutation test to calculate the enrichment score (ES) within predefined gene sets from the Hallmark database. Enrichment scores were normalized regarding gene set size and presented as normalized enrichment score (NES). The results of the GSEA analysis for the ranked log2 fold change values of CS patients vs. control group are presented in Figure 7, where the twenty hallmark database groups with the highest absolute NES values are included. Within the gene ranks column, each vertical line represents one gene and its position depends on the logFC value (around 0 are the genes with high logFC value, on which CS had a stimulating effect, thus are enriched genes. On the right side, around 18285, there are genes whose expression is due to CS action is lowered and had a low logFC value and these are depleted genes). Despite a different methodological approach, the GSEA analysis presents relatively similar groups as shown in the analysis of ontological groups by DAVID. These groups concern apoptosis and immunological processes. The analysis regarding the position of single genes on the gene rank scale and consequently the NES values of individual groups, indicates that CS stimulates expression of genes in the immunity-related processes hallmark group, including “Complement system” and “Interferon gamma response”. Likewise, genes are expressed which are involved in inflammation, including the “Inflammatory response” and “TNF alpha via NFkB” pathways. Genes involved in the regulation of apoptosis are also intensely stimulated by CS. The analysis of enrichment within the hallmark apoptosis group is characterized by a positive value of NES = 2.66. Interestingly, the GSEA analysis also showed a significant increase in the expression of genes related to proliferation regulation belonging to the following sets of hallmark database: “mitotic spindle” (NES = 2.59) and “Pi3K_AKT_MTOR Signaling” (NES = 3.42), including genes with a very high logFC value, as shown in Figure 7.

**Figure 8:**
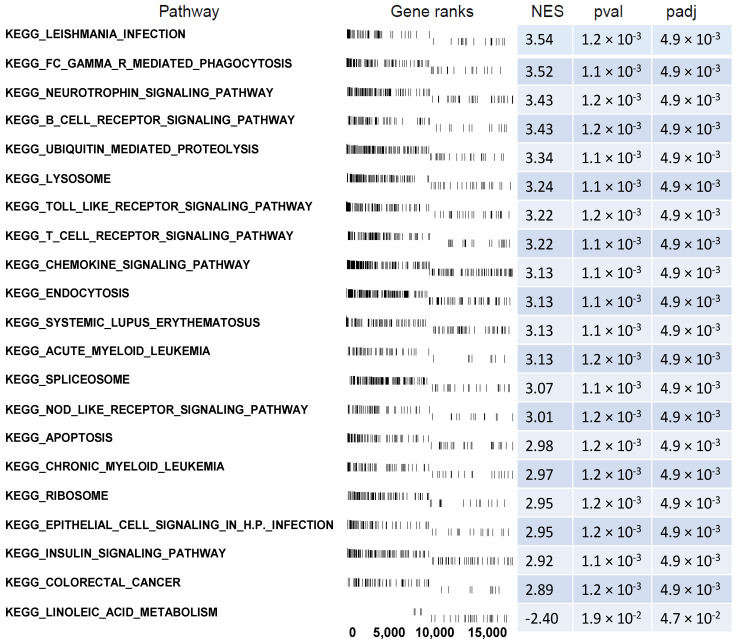
Gene set enrichment analysis in CD34+ hematopoietic progenitor cells from CS patients using KEGG gene sets. A list of significantly enriched gene sets with appropriate gene ranks, normalized enrichment score (NES), *p* values (pval), and *p* values after FDR correction (padj) is displayed.

On the same DEGs, we also performed gene set enrichment analysis within predefined gene sets from the Kyoto Encyclopedia of Genes and Genomes (KEGG) database. For this analysis, we included both the genes above and below the cut-off of |fold change| = 1.5 and *p* = 0.05. First, the fold values of all of the genes were log2 transformed and ranked based on the log2 fold change. These values were used to calculate the Enrichment Score (ES, a permutation test run 1000 times) within predefined gene sets from the KEGG database. The core-enriched genes for the GSEA-enriched pathways in the CD34+ hematopoietic progenitor cells from CS patients are shown in a ridgeline plot (Figure 8). Some of the top enriched terms are related to the cellular responses to GCs, and include the overrepresented “Apoptosis”, “Endocytosis”, and “Ubiquitin mediated proteolysis”.

**Figure 9:**
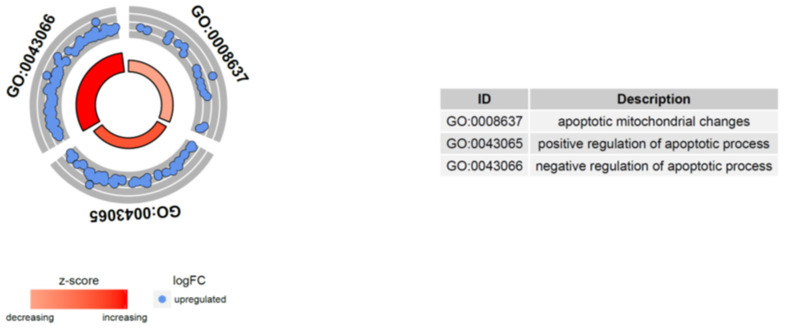
Bioinformatics analysis of three enriched gene ontology (GO) groups related to biological processes regulating apoptosis in CD34+ hematopoietic progenitor cells from CS patients and controls. The processes disturbed in CD34+ hematopoietic progenitor cells from CS patients are selected and presented in the table on the right side of the pie chart. Each dot represents a single gene, which is upregulated due to GC activity in cells. Positive values of Z-score mapped on a red color scale are presented inside the graph.

DAVID and GSEA analyses indicated potent regulation by GCs of genes related to apoptosis; therefore, in the next part, we analyzed specific genes belonging to the following ontological groups: Apoptotic mitochondrial changes (GO: 0008637), Positive regulation of apoptotic process (GO: 0043065), and Negative regulation of apoptotic process (GO: 0043066) (Figure 9). The fold change values of the genes contained in the mentioned groups were used to calculate the Z-score, which indicates whether the process decreased (negative value) or increased (positive value), and are presented as circular scatter plots. The Z-score was calculated automatically using the GO plot library. All of the analyzed ontological groups were characterized by a positive Z-score, confirming the stimulating effect exerted by GCs on apoptosis-related processes. (Figure 9).

**Figure 10:**
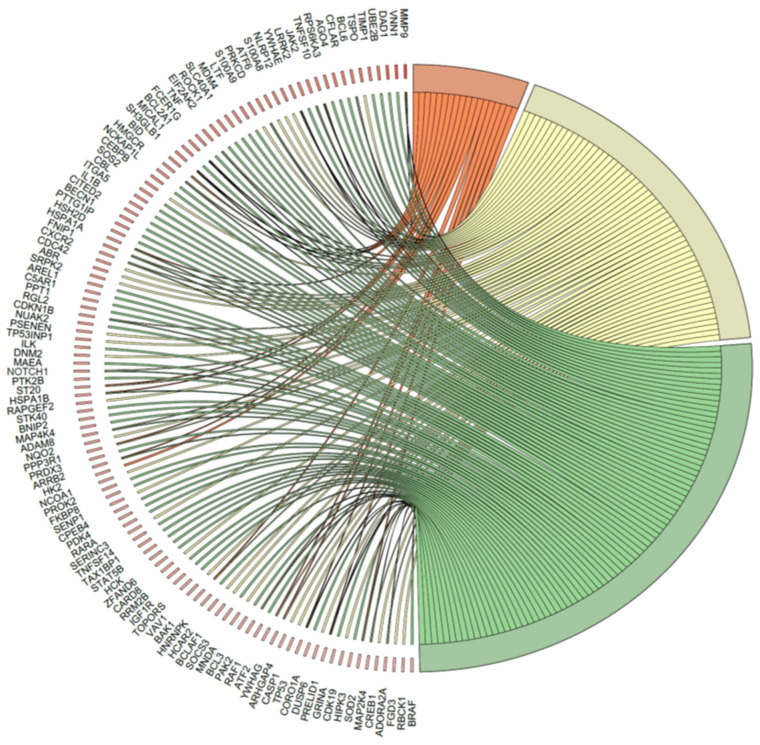

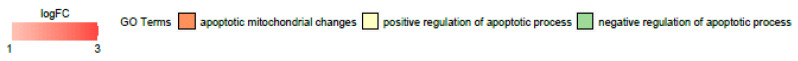
The relationship between particular genes belonging to analyzed GO terms (Apoptotic mitochondrial changes, Positive regulation of apoptotic process, Negative regulation of apoptotic process) is mapped in a circos plot. All of the genes in CD34+ hematopoietic progenitor cells from CS patients were upregulated compared to controls. LogFC values and gene symbols are shown on the left side of the graph. The level of expression for each gene is marked using a red color (fold change one to three). Ribbons connecting areas of the circus plots also indicate shared genes between GO terms.

Due to the ambiguous nature of the structure of the GO database, single genes can often be assigned to several ontological terms. For this reason, the relationship between genes and GO terms related to the apoptosis process and its positive and negative regulation were mapped with a circus plot, with visualization of logFC values and gene symbols (Figure 10). All of those genes were upregulated in CD34+ hematopoietic progenitor cells from CS patients compared to controls. The strongest upregulated genes from the examined ontological groups included, among others: *MMP9* (Matrix Metallopeptidase 9), *DAD1* (Defender Against Cell Death 1), *TIMP1* (Tissue Metallopeptidase Inhibitor 1), *BCL6* (B-cell lymphoma 6), *TNFSF10* (TNF Superfamily Member 10), and *TNF* (tumor necrosis factor). Analogous to the David analysis with circosplot visualization, most of the genes displayed in “Apoptosis” and “Regulation of apoptotic process” were upregulated. This result confirms the stimulatory effect of glucocorticoids on apoptosis activation. This result confirms the stimulatory effect of GCs on apoptosis activation in hematopoietic progenitor cells.

### 2.6. Microarray Analysis of miRNA Expression Changes in CD34+ Hematopoietic Progenitor Cells from CS Patients

**Figure 11:**
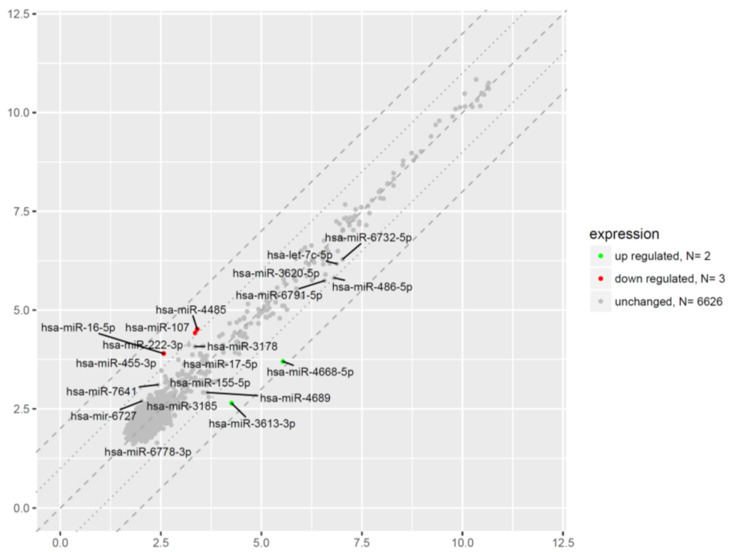
The scatter plot of global miRNA expression in CD34+ hematopoietic progenitor cells from CS patients when compared to their controls. Red points correspond to downregulated genes (at least two-fold change, *p* < 0.05), green points indicate upregulated genes (at least two-fold change, *p* < 0.05). The graph also contains names of the miRNA with the largest change in expression. On the horizontal axis (*x*-axis) are plotted data from the CS patient group in this study. On the vertical axis (*y*-axis) are plotted data from the healthy subject group, which serves as the control group in this study.

In order to gain an expression profile of miRNAs in peripheral blood-derived CD34+ hematopoietic progenitor cells from CS patients, a microarray was used to identify the differentially expressed miRNA. We applied a stringent filtering approach that compared CS patients with healthy volunteers (absolute fold change > 2.0). There were a total of five aberrantly expressed miRNAs in hematopoietic progenitor cells from CS patients (Figure 11). The significantly differentially expressed miRNAs characterized by high fold change (FC) values ranged from 3.07 to 3.57 for upregulated miRNAs, and for downregulated genes from −2.02 to −2.50. The two miRNAs that were significantly upregulated in CS patients were miRNA-3613-3p and miRNA-4668-5p. The highest expression was observed in the case of miRNA-4668-5p transcript with a nearly four-fold difference in the expression compared to healthy controls. The three miRNAs that were significantly downregulated in CS patients are miRNA-107, miRNA-16-5p, and miRNA-4485. The lowest expression was observed in the case of miRNA-16-5p transcript with a nearly three-fold difference in the expression compared to healthy controls.

In an effort to better understand the molecular function or biological process difference between miRNA profiles in CS patients groups in this study, we analyzed the Gene Ontology (GO) of miRNAs target genes. In the group of the significantly changed miRNA expression, the GO analysis identified the involvement of these miRNAs in various biological processes, such as miRNA-mediated gene silencing and miRNA-mediated gene silencing by inhibition of translation (miR-16, miR-107), positive regulation of translation (miR-16), negative regulation of I-kappaB kinase/NF-kappaB signaling (miR-16), cellular response to glucose stimulus (miR-16), negative regulation of glucose import (miR-107), positive regulation of gluconeogenesis (miR-107), negative regulation of cytokine production involved in inflammatory response (miR-16), positive regulation of connective tissue replacement (miR-16), positive regulation of apoptotic process (miR-16), positive regulation of intrinsic apoptotic signaling pathway (miR-16), positive regulation of necroptotic process (miR-107), and cellular response to hydrogen peroxide (miR-107).

**Figure 12:**
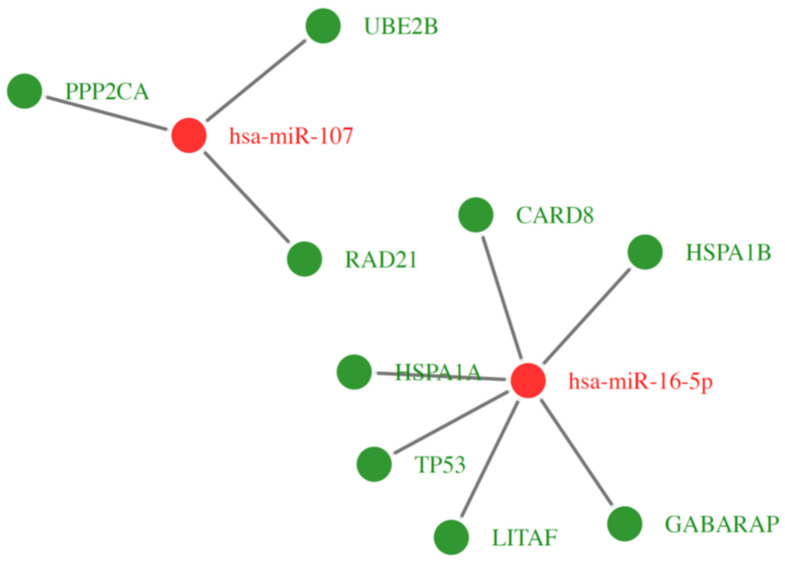
This diagram shows significantly downregulated miRNAs (at least two-fold) in CD34+ hematopoietic stem and progenitor cells from CS patients. Target genes are assigned to each miRNA with a marked change in expression (at least two-fold) (green indicates upregulation; red indicates downregulation).

On the other hand, in the group of the significantly downregulated miRNA transcripts in CD34+ hematopoietic progenitor cells from CS patients, the GO analysis identified miRNAs which regulate various processes related to programmed cell death, including induction of apoptosis and regulation of the apoptotic process. Figure 12 shows the two most downregulated miRNAs and their targeted genes with changes in their expression in CS patients when compared to their controls in this study. In particular, the most downregulated, miR-16 transcript (around three fold), can participate in the expression regulation of mRNA sequence encoding several protein molecules involved in crucial cellular mechanisms related to programmed cell death and its control. Among them is upregulated Caspase Recruitment Domain Family Member 8 (CARD8). CARD8 is involved in pathways leading to activation of caspases and nuclear factor kappa-B (NFKB). This protein was found to be a component of the NLRP3 inflammasome, a main protein complex that plays a role in the activation of pro-inflammatory caspases. It is also proposed that CARD8 acts as an adaptor molecule that negatively regulates NFKB activation, CASP1-dependent IL-1B secretion, and finally mediates apoptosis in response to CARD8 inflammasome activation. One of the activated genes was Lipopolysaccharide Induced TNF Factor (LITAF). This gene encodes lipopolysaccharide-induced TNF-alpha factor, which is a DNA-binding protein and can mediate the TNF-alpha expression by direct binding to the promoter region of the TNF-alpha gene. The transcription of this gene is induced by tumor suppressor protein p53 (TP53) and has been implicated in the p53-induced apoptotic pathway. It also may play a synergistic role with STAT6 in the cell nucleus in regulating the expression of various cytokines, such as TNF-alpha, CCL2, CCL5, CXCL1, IL-1A, and IL-10. The RNA sequence of the above-mentioned TP53 molecule was also upregulated under the significant control of the downregulated miR-16 in our study. It acts as a tumor suppressor in many cell types and may induce growth arrest or apoptosis depending on the physiological circumstances and cell type. Apoptosis induction through TP53 seems to be mediated either by stimulation of BAX expression, or by repression of BCL-2 expression. The relevant data on Gene Ontology (GO) Biological Process for the most upregulated genes involved in apoptosis process control, together with other types of cell death and inflammatory state, are presented in Table 1.

## 3. Discussion

Glucocorticoids (GCs) are hormones produced by the adrenal cortex. GCs participate in many important physiological processes, including metabolism, inflammation, immunity, and cellular stress. GCs provide many effects on immune system cellular components, and their impact on mature white blood cells is very important, including the influence on their amounts and functions. The concept that GCs are directly involved in the hematopoiesis of patients with hypercortisolism prompted us to analyze the influence of GCs on human hematopoiesis in patients with Cushing’s Syndrome. Our previous studies on human peripheral blood-derived CD34+-enriched hematopoietic stem and progenitor cells suggested that other hormones, such as thyroid hormones or growth hormones, released in an excessive manner due to specific endocrinopathies could play a role in the regulation of the growth, survival, and apoptosis of human early hematopoietic stem cells [6,7,8], thus potentially affecting the peripheral blood cell counts, abnormalities of which are observed concurrently in different endocrine dysfunctions in humans.

Glucocorticoids exert their effects through complicated mechanisms; however, it is predominantly via the GC glucocorticoid receptor (GR). The genomic function of GCs is mainly to bind to specific GRs in the cytoplasm to form complexes and transfer to the nucleus, where it regulates the transcriptional activity of GC response genes. The GR acts as a classical transcription factor that promotes or suppresses the transcription of target genes by binding to specific DNA sequences known as glucocorticoid-response elements (GREs). In some cases, GR represses transcription by binding to negative GREs (nGREs). In addition, ligand-bound GR can be recruited to specific genomic sites via protein–protein interactions with other DNA-bound transcription factors. GCs also exert genomic effects by interfering with the activity of several transcription factors and diverse signaling molecules [25]. As GR expression is a key target that influences the effectiveness of GC-dependent genomic effects, the analysis of its expression in circulating HSPCs was highly contributory. In the present study, we used qRT-PCR to detect GR alpha protein expression in hematopoietic progenitors of patients with endogenous hypercortisolism. Both groups, the examined patients and the control group, expressed protein for the examined GR alpha as the most frequent form found in human cells. We reported here for the first time that CD34+-enriched HSPCs from patients with CS expressed GR alpha at protein levels (Figure 1). It has been documented that expression of GR was present in peripheral blood lineage-committed hematopoietic cell subsets, including lymphoid and myeloid cell lineages [26,27], or other stem cell types, such as mesenchymal stem cells (MSC) [28], neural stem cells (NSC) [29] or even embryonic stem cells (ESC) [30] and periodontal ligament stem cells (PLSC) [31]. Notably, only a few studies have investigated expression of GR in hematopoietic stem cells of different origin, including embryonic [32] or cord blood-derived [33]. Moreover, we observed a more than 50% upregulation of GR alpha in the PB-derived CD34+ progenitor cells from patients with endogenous hypercortisolism compared to healthy subjects (*p* < 0.01). These findings may indicate that the abundant presence of GCs may augment the expression of GR alpha in circulating CD34+-enriched HSPCs. Likewise, similar to our results, were those reported by Vidović et al., who observed that glucocorticoid receptor expression positively correlated with serum cortisol concentration in healthy subjects not affected by any of known endocrinopathies [34]. These observations indicate that the expression of GR in hematopoietic stem and progenitor cells may be dependent on the chronic GC status in peripheral blood.

Regulation of cell survival and apoptosis is regulated by the Bcl-2 family that consists of pro- and anti-apoptotic proteins. Previously, several in vitro and animal experiments have demonstrated that pro- and anti-apoptotic members of the Bcl-2 family are involved in GC-induced apoptosis in white blood cells, including lymphocytes and neutrophils, the two main mature populations of WBC circulating in peripheral blood. For example, the in vivo experiments have shown that GCs are involved in regulating T and B lymphocyte apoptosis via genomic mechanisms [35,36]. GCs can reduce peripheral blood lymphocyte counts. The peripheral blood lymphocyte count decreased significantly after short- or long-term application of GCs in animal experiments. Unfortunately, the mechanism of pro-apoptotic signal transduction has not been entirely clarified. Moreover, it is challenging to apply results obtained in vitro as well as in vivo to human physiology. In our study, we demonstrated a significant increase in the percentage of apoptotic CD34+ HSPCs in both early and late phases of apoptosis collected from CS patients compared with healthy volunteers (Figure 2). This finding is in accordance with previously published data showing that incubation with non-physiological concentrations of GCs induced programmed cell death in bone marrow-derived lymphocytes [37].

Next, we determined whether genes with cell death/survival functions are expressed and are differentially modulated by GCs in PB-derived CD34+-enriched HSPCs from patients with chronic hypercortisolism due to endogenous CS. Therefore, we investigated the expressions of pro-apoptotic BAX gene anti-apoptotic BCL-xL and BCL-2 genes and at the mRNA level in the CD34+-enriched HSPCs. We established a significant relationship between the augmented GC production in the patients with CS and expression of the pro- and anti-apoptotic genes. Here, the hematopoietic progenitors collected from peripheral blood of patients with adrenal dysfunction presented a strongly increased expression of the pro-apoptotic gene BAX at the mRNA level. Cells harvested from patients suffering from ACTH-independent CS presented increased expressions of mRNA for the BAX gene of around 35% compared with the healthy controls. We noted an even more marked growth in the expressions of the pro-apoptotic BAX gene, with a nearly 60% increase in the levels in patients suffering from ACTH-dependent CS compared with the healthy controls (Figure 3a). The mRNA for the BAX gene is expressed at high levels during the period when programmed cell death normally takes place in different cells. In parallel, CD 34+ cells harvested from CS patients presented increased expressions of mRNA for BCL-2 and BCL-xL; thus, over-expressed anti-apoptotic genes could play a role in the homeostatic balance of programmed cell death observed in our patients with CS (Figure 3b,c, respectively). Similarl to our results, the increased expression of BCL-2 and BCL-xL was also observed in other lineage-committed hematopoietic cells, such as effector T cells, following GC treatment in vitro [38,39]. The upregulation of the both anti-apoptotic molecules Bcl-2 and Bcl-xL in activated T cells was proposed to be an important mechanism of the T cells’ reduced sensitivity to GC-induced apoptosis [39]. The BCL-2 family comprising the main regulatory factors that control the intrinsic pathway of cell death has also been shown to be uniquely regulated in stem cells [40]. Notably, different genes with up- or down-regulated expression in GC-induced apoptosis including c-myc, tdag8, dig2, Bim, and PUMA have been described by different reports [36,41]. These data suggest that GCs may promote the apoptosis of early hematopoietic progenitors in endogenous CS patients and that glucocorticoid signaling has an important, but understudied, role in HSPC life-or-death decisions.

Therefore, we decided to verify the potential associations between naturally increased systemic levels of GC and the expression of apoptosis-related genes in CD34+ cells from CS patients by comparing the genome-wide gene expression profiles of these cells collected from all the groups. We were interested especially in genes involved in biological processes concerning induction and regulation of programmed cell death, extrinsic/intrinsic apoptotic signaling pathways, regulation of apoptotic signaling pathways, apoptosis-related nuclear and mitochondrial changes, cell-type specific apoptotic processes, and other similar processes related to programmed cell death. Microarray analysis showed that expression of 1152 genes was upregulated at least two-fold and 103 genes were downregulated at least two-fold in the CS group compared to the control group (Figure 4). A more detailed analysis of selected pathways related to apoptosis revealed at least seven pathways from the GO BP Direct database that were related to apoptosis and its control via positive or negative regulation, including pathways denoted as: Apoptotic process and Apoptotic signaling pathway, Intrinsic apoptotic signaling pathway, Negative regulation of apoptotic process, Positive regulation of apoptotic process, Regulation of apoptotic process, and Apoptotic mitochondrial changes (Figure 5).

Exploring the effects of chronic endogenous hypercortisolism on microRNA expression in HSPC, the microarray analysis carried out in our laboratory showed that there were in total five aberrantly expressed miRNAs in hematopoietic progenitor cells from CS patients (Figure 11). The three miRNAs that were significantly downregulated in CS patients were miRNA-107, miRNA-16-5p, and miRNA-4485. The other two miRNAs that were significantly upregulated in CS patients were miRNA-3613-3p and miRNA-4668-5p. Further bioinformatic analysis revealed that downregulated miRNA sequences may be engaged in several biological processes, which might regulate programed cell death through positive regulation of apoptotic process (miR-16), positive regulation of intrinsic apoptotic signaling pathway (miR-16), positive regulation of necroptotic process (miR-107), and cellular response to hydrogen peroxide (miR-107) in CD34+ hematopoietic stem and progenitor cells from CS patients. The data obtained in this study are in line with the results of previous reports describing other unique microRNA sequences with potential to control apoptotic process. These microRNAs, specifically regulated by GCs or their receptors, are able to promote apoptosis, such as miR-22 [42], or inhibit apoptosis, such as miR-124 [43].

It was reported that induction of T-cell apoptosis by GCs requires DNA-binding dependent transcriptional regulation, presumably of pro-apoptotic genes such as Bim and PUMA [44], and is mediated via the intrinsic apoptotic pathway [45]. In our study, downregulated miRNA involved in the regulation of cellular functions, including apoptosis via the positive regulation of intrinsic apoptotic signaling pathway, was indeed found to bemiR-16. The miRNAs are key checkpoint regulators in a wide variety of biological processes, and dysregulation of these small noncoding RNAs can alter cellular homeostasis and therefore may contribute to disease-related metabolic and cellular abnormalities, including apoptosis dysregulation in hematopoietic stem and progenitor cells present in bone marrow and peripheral blood.

## 4. Materials and Methods

### 4.1. Human Subjects

We enrolled 33 patients with hypercortisolism due to newly diagnosed endogenous Cushing’s Syndrome (CS) in the Department of Endocrinology and Metabolic Diseases of Pomeranian Medical University in Szczecin, Poland. The patients were classified into groups according to the diagnosis. In the group with hypercortisolism due to adrenocortical tumors, there were 16 subjects (10 females), who were assigned to ACTH-independent Cushing’s Syndrome (AID-CS). In the group with hypercortisolism due to pituitary tumors, there were 17 subjects (10 females), who were assigned to ACTH-dependent Cushing’s Syndrome (AD-CS). The control group consisted of 35 age-matched participants (20 females) with no symptoms or signs of CS (defined as the absence of typical clinical features of CS). Importantly, patients with known infectious or active inflammatory disorders did not undergo biochemical evaluation for hypercortisolemia until resolution of symptoms, and thus they were not included in the study. The diagnosis of initial or recurrent CS was based on criteria defined by the Endocrine Society guidelines. All of the enrolled subjects underwent a complete medical examination for statistical analyses. Data regarding medical history, current drug use, and smoking status were collected based on laboratory data, pathology tests, and other information, with a particular focus on heart and vascular conditions and existing arterial hypertension. All procedures were approved by the local ethics committee. Moreover, informed consent was given in every case.

### 4.2. Blood Sample Collection

Venous blood samples (~15 mL) collected in EDTA tubes were centrifuged (2000 rpm, 4 °C, 10 min) and the plasma was stored at −20 °C to −80 °C until assayed. Next, the red blood cells were lysed using BD Pharm Lyse lysing buffer (BD Biosciences, San Jose, CA, USA) for 15 min at room temperature for the isolation of peripheral blood nuclear cells (PBNCs).

### 4.3. Cell Isolation

Next, the obtained PBNCs fractions were depleted of adherent cells and T lymphocytes (A-T-MNCs) as described in [46]. The isolated fraction was enriched with CD34+ cells using a CD34+ isolation MiniMACS kit (Miltenyi Biotech, Sunnyvale, CA, USA) according to the manufacturer’s protocol. Isolated CD34+ cells were next analyzed by flow cytometry to evaluate the efficiency of the CD34-positive sorting. The purity of the collected CD34+-enriched hematopoietic progenitor cells (HPCs) was over 95%. The viability of cells was assayed by the trypan blue exclusion test. The cells were counted using a standard hemocytometer.

### 4.4. Flow Cytometry

The CD34+ cells were quantitatively analyzed for the expression of glucocorticoid receptor (GR) alpha (GRα) (USBiological, Salem, MA, USA) using flow cytometry. Briefly, CD34+ cells were permeabilized by adding 0.25 mL of CytoFix/CytoPerm solution per tube and incubating the tubes in the dark at 4 °C for 15 min. Two milliliters CytoFix/CytoPerm wash buffer then was added followed by centrifugation (10 min at 4 °C). The resulting supernatant was discarded, and cells were resuspended in 1 mL PBS. Next, permeabilized cells were incubated (30 min) with appropriately diluted anti-human fluorochrome-conjugated monoclonal antibodies against specific antigens, including GRα, CD34 (BD Biosciences, San Jose, CA, USA), and CD45 (BD Biosciences, San Jose, CA, USA). The cells were washed twice in ice-cold PBS, resuspended in 1% paraformaldehyde and analyzed in a flow cytometer (LSRII, BD Biosciences, San Jose, CA, USA) using the BD FACSDiva software (BD Biosciences, San Jose, CA, USA). Importantly, the purchased antibody did not detect GR beta.

### 4.5. Apoptosis Detection

The level of spontaneous apoptosis in CD34+ cells circulating in PB of enrolled patients and controls was measured using two different methods. The Annexin V-FITC Apoptosis Detection Kit II (BD Biosciences, San Jose, CA, USA) was used for the detection of the early stage of apoptosis. To detect the late stage of apoptosis, the collected CD34+ cells were analyzed with the terminal deoxynucleotidyl transferase dUTP nick end labeling (TUNEL) assay using the APO-Direct Kit (BD Biosciences, San Jose, CA, USA). Both kits were used according to the manufacturer’s instructions.

### 4.6. RNA Isolation and Gene Expression Analysis

Total mRNA was isolated from CD34+ cells using the RNeasy Mini Kit (Qiagen, Valencia, CA, USA). Subsequently, mRNA was reverse transcribed using the First Strand cDNA Synthesis Kit (Fermentas International Inc., Burlington, ON, Canada). Quantitative assessment of BCL-2, BCL-XL, and BAX mRNA levels was performed using real time QRT-PCR carried out on a Bio-Rad CFX96 Real-Time PCR Detection System (Bio-Rad Inc., Hercules, CA, USA). The 25-µL reaction mixture contained 12.5 µL of SYBR Green PCR Master Mix, 10 ng of cDNA template, and one pair of the primers listed in Table 2. The relative quantification value of the target gene was normalized to the endogenous control gene of beta-2 microglobulin (BMG) and expressed as 2^∆Ct^, where ∆Ct = [Ct of BMG] − [Ct of target gene].

### 4.7. RNA and MiRNA Isolation for Microarrays

Total RNA enriched in miRNAs was isolated from peripheral blood CD34+ cells collected from all groups in the study using the mirVanaTM miRNA Isolation Kit (Thermo Fisher, Waltham, MA, USA), following the manufacturer’s instructions. The concentration and quality of the obtained RNA were assessed by an Epoch spectrophotometer (Biotek, Winooski, VT, USA). For subsequent miRNA and whole transcriptome microarray analysis, total RNA enriched in miRNAs isolated from CD34+ cells samples were pooled to generate one representative sample per group.

### 4.8. Affymetrix GeneChip Whole Transcriptome Microarray

A sense-strand cDNA generated from the total RNA using an Ambion WT Expression Kit (Thermo Fisher Scientific, Waltham, MA, USA) was fragmented and labelled using the GeneChip WT Terminal Labelling Kit (Affymetrix, Santa Clara, CA, USA) and next hybridized onto an Affymetrix Human Gene 2.1 ST Array Strip. The hybridization and subsequent fluidics and scanning steps were carried out with an Affymetrix GeneAtlas System with designated software (Affymetrix, Santa Clara, CA, USA).

### 4.9. Affymetrix GeneChip miRNA Microarray

The procedure using previously described methods [47,48] started with a poly(A) tailing reaction followed by ligation of the biotinylated signal molecule to the target RNA. The next step was sample hybridization onto an Affymetrix miRNA 4.1 Array Strip (Affymetrix, Santa Clara, CA, USA). The last step was streptavidin-PE addition and array scanning with Affymetrix GeneAtlas system (Affymetrix, Santa Clara, CA, USA).

### 4.10. Microarrays Data Analysis

Analysis of miRNA and whole transcriptome microarray data was performed using BioConductor software based on the statistical R programming language. The Robust MultiArray Average (RMA) normalization algorithm implemented in the “Affy” library was used for normalization, background correction, and calculation of the expression levels of all of the examined genes and miRNAs.

For whole transcriptome microarray, the normalized data set was merged with an annotated data frame object from the BioConductor “oligo” package, leading to a complete gene data table. The selection criteria for significantly changed gene expression were based on the expression fold difference higher than |2|. Functional annotation clustering of differentially expressed genes was performed using the DAVID database and is shown as a bubble plot. The cut-off criteria for generation of bubble plot were as follows: *p*-value < 0.5, adjusted method = Benjamini, and minimal number of genes per group = 100. Groups of genes fulfilling the mentioned criteria are presented in a graph, in which the bubble size indicates the number of genes represented in the corresponding annotation.

For the miRNA microarray, normalized data were combined with the “pd.mirna.4.1” description file, containing, among others, names, types, and sequences of miRNAs. Differential expression was determined by applying the linear models for microarray data implemented in the “limma” library. Normalized miRNA expression datasets were visualized on scatter plots with relation to determined cut-off criteria (fold change higher than |2|). A list of experimentally validated miRNA target genes was downloaded from miRTarBase, a database of mRNA–target interactions. Only targets for differentially expressed miRNA were subtracted from the whole human miRNA–target dataset. A target gene list from each of the comparisons was subjected to functional annotation and clusterization using DAVID (database for annotation, visualization, and integrated discovery). Target symbols of differentially expressed miRNA were uploaded to DAVID by the “RDAVIDWebService” BioConductor library, where targets were assigned to relevant Gene Ontology (GO) terms.

Patients with Cushing’s Syndrome were prospectively enrolled into the Cushing’s Syndrome subgroup of longitudinal study of endocrine disorders cohort, and analyses of microRNA datasets from the Cushing’s Syndrome subgroup, augmenting in number of enrolled patients within the whole period of large study, were used to study different aspects of microRNA expression changes in course of Cushing’s Syndrome. Thus, the microRNA data, at least for some of the patients, analyzed in different groups of this study, have been published elsewhere [23], using different methods of results comparison analysis.

### 4.11. Statistical Methods

Differences in the values of the quantitative parameters were compared between groups by unpaired Student *t*-test with Welch’s correction; for non-parametric tests, values were compared using the Mann–Whitney test. A *p* value of <0.05 was considered statistically significant.

## 5. Conclusions

In conclusion, we revealed here a direct cause-and-effect association between adrenal disorders with excessive production of glucocorticoids and human hematopoiesis. These findings provide novel evidence to support the hypothesis that elevated GC concentrations, such as those resulting from Cushing’s Syndrome, have the potential to impair cellular function and hematopoiesis quality. Particularly, our study extents previous findings concerning GC-induced apoptosis of stem cells, which reflects the very complex nature of this process in humans in vivo and is worthy of further investigation. Especially, the current literature on the effects of glucocorticoid-based induction of apoptosis in human hematopoiesis-related cells is contradictory, in part due to the wide variety of gene networks affected by GCs-GR axis. Moreover, the sensitivity of HSPCs to GC action may depend on their maturation level and activation status. Therefore, our observations may help to improve the understanding of the interactions between GC function and human hematopoiesis and provide a basis for designing a novel view for diagnosis and therapeutic interventions in Cushing’s Syndrome.

## Figures and Tables

**Table 1:** List of the significantly downregulated two miRNA in CD34+ cells from CS patients and their target genes assigned to each miRNA with a marked change in expression (at least two-fold upregulation) and gene function according to GO Biological Processes database.

Gene Symbol	Gene Name	Gene Function According to GO Biological Processes Database	miRNA
CARD8	Caspase Recruitment Domain Family Member 8	programmed cell death (GO:0012501), regulation of apoptotic process (GO:0042981), regulation of I-kappaB kinase/NF-kappaB signaling (GO:0043122), negative regulation of I-kappaB kinase/NF-kappaB signaling (GO:0043124), negative regulation of NF-kappaB transcription factor activity (GO:0032088), activation of cysteine-type endopeptidase activity involved in apoptotic process (GO:0043280), positive regulation of interleukin-1 beta production (GO:0032731), inflammatory response (GO:0006954), negative regulation of NLRP3 inflammasome complex assembly (GO:1900226)	miRNA-16
LITAF	Lipopolysaccharide Induced TNF Factor	regulation of cytokine production (GO:0001817), regulation of macrophage cytokine production (GO:0010935), response to lipopolysaccharide (GO:0032496), cellular response to lipopolysaccharide (GO:0071222), positive regulation of I-kappaB kinase/NF-kappaB signaling (GO:0043123), negative regulation of NIK/NF-kappaB signaling (GO:1901223)	miRNA-16
TP53	Tumor Suppressor Protein P53	apoptotic process (GO:0006915), regulation of apoptotic process (GO:0042981), positive regulation of apoptotic process (GO:0043065), intrinsic apoptotic signaling pathway (GO:0097193), positive regulation of intrinsic apoptotic signaling pathway (GO:2001244), regulation of fibroblast apoptotic process (GO:2000269), cardiac muscle cell apoptotic process (GO:0010659), positive regulation of cardiac muscle cell apoptotic process (GO:0010666), programmed cell death (GO:0012501), intrinsic apoptotic signaling pathway in response to DNA damage by p53 class mediator (GO:0042771), regulation of neuron apoptotic process (GO:0043523), positive regulation of neuron apoptotic process (GO:0043525), regulation of thymocyte apoptotic process (GO:0070242), positive regulation of thymocyte apoptotic process (GO:0070245)	miRNA-16
HSPA1A	Heat Shock Protein Family A (Hsp70) Member 1A	regulation of cell death (GO:0010941), negative regulation of apoptotic process (GO:0043066), negative regulation of mitochondrial outer membrane permeabilization involved in apoptotic process (GO:1901029), negative regulation of extrinsic apoptotic signaling pathway in absence of ligand (GO:2001240), cellular response to oxidative stress (GO:0034605), negative regulation of endoplasmic reticulum stress-induced intrinsic apoptotic signaling pathway (GO:1902236), negative regulation of cell growth (GO:0030308), regulation of protein ubiquitination (GO:0031396), negative regulation of protein ubiquitination (GO:0031397), positive regulation of tumor necrosis factor-mediated signaling pathway (GO:1903265)	miRNA-16
HSPA1B	Heat Shock Protein Family A (Hsp70) Member 1B	negative regulation of apoptotic process (GO:0043066), negative regulation of extrinsic apoptotic signaling pathway in absence of ligand (GO:2001240), negative regulation of cell death (GO:0060548), cellular response to steroid hormone stimulus (GO:0071383), cellular response to oxidative stress (GO:0034599), positive regulation of NF-kappaB transcription factor activity (GO:0051092), positive regulation of tumor necrosis factor-mediated signaling pathway (GO:1903265), positive regulation of interleukin-8 production (GO:0032757)	miRNA-16
GABARAP	GABA Type A Receptor-Associated Protein	apoptotic process (GO:0006915), extrinsic apoptotic signaling pathway via death domain receptors (GO:0008625), autophagy (GO:0006914), autophagy of mitochondrion (GO:0000422), autophagosome assembly (GO:0000045), macroautophagy (GO:0016236), positive regulation of proteasomal ubiquitin-dependent protein catabolic process (GO:0032436), regulation of Rac protein signal transduction (GO:0035020)	miRNA-16
PPP2CA	Protein Phosphatase 2 Catalytic Subunit Alpha	apoptotic process (GO:0006915), response to organic substance (GO:0010033), negative regulation of cell growth (GO:0030308), negative regulation of epithelial to mesenchymal transition (GO:0010719)	miRNA-107
RAD21	Cohesin Complex Component	apoptotic process (GO:0006915), cellular response to DNA damage stimulus (GO:0006974), double-strand break repair (GO:0006302), replication-born double-strand break repair via sister chromatid exchange (GO:1990414)	miRNA-107
UBE2B	Ubiquitin Conjugating Enzyme E2 B	apoptotic process (GO:0006915), negative regulation of apoptotic process (GO:0043066), response to xenobiotic stimulus (GO:0009410), cellular response to DNA damage stimulus (GO:0006974), DNA repair (GO:0006281), ubiquitin-dependent protein catabolic process (GO:0006511), protein polyubiquitination (GO:0000209), protein monoubiquitination (GO:0006513)	miRNA-107

**Table 2:** List of primers for apoptosis detection in CD34+ cells used in this study.

No.	Gene Name	Gene Symbol	Primer Direction	Primer Sequence
1	B-cell CLL/lymphoma 2	*BCL* *-2*	Sense Antisense	GCC GGT TCA GGT ACT CAG TCA T CAT GTG TGT GGA GAG CGT CAA
2	B-cell lymphoma-extra large	*BCL-XL*	Sense Antisense	CTC AGC GCT TGC TTT AC CGC ACA GCA GCA GTT TGG
3	BCL2-associated X protein	*BAX*	Sense Antisense	GTT GCG GTC AGA AAA CAT GTC GCC GCC GTG GAC ACA
4	Beta-2-microglobulin	*BMG*	Sense Antisense	AAT GCG GCA TCT TCA AAC CT TGA CTT TGT CAC AGC CCA AGA TA

## Data Availability

The original contributions presented in the study are included in the article. Further inquiries can be directed to the corresponding authors.
